# Contrastive multimodal deep learning for survival prediction in grade 2/3 gliomas

**DOI:** 10.1093/jncics/pkag039

**Published:** 2026-04-15

**Authors:** Peiying Hua, Chun-Chieh Lin, Travis Fenlon, Xiaoying Liu, Saeed Hassanpour

**Affiliations:** Department of Biomedical Data Science, Geisel School of Medicine at Dartmouth, Hanover, NH, United States; Department of Pathology and Laboratory Medicine, Dartmouth-Hitchcock Medical Center, Lebanon, NH, United States; Department of Pathology and Laboratory Medicine, Dartmouth-Hitchcock Medical Center, Lebanon, NH, United States; Department of Pathology and Laboratory Medicine, Dartmouth-Hitchcock Medical Center, Lebanon, NH, United States; Department of Biomedical Data Science, Geisel School of Medicine at Dartmouth, Hanover, NH, United States; Department of Epidemiology, Geisel School of Medicine at Dartmouth, Hanover, NH, United States; Department of Computer Science, Dartmouth College, Hanover, NH, United States

## Abstract

**Background:**

Accurate survival prediction for grade 2/3 glioma patients remains challenging due to tumor biological heterogeneity and limitations of current prognostic methods that rely on single-modality data.

**Methods:**

We developed a multimodal deep learning framework integrating histopathology whole-slide images, somatic mutations, and clinical-demographic data. A 3-stage training pipeline combined contrastive learning with survival-specific optimization to align cross-modal representations. The framework was trained on 498 grade 2/3 glioma patients from The Cancer Genome Atlas and evaluated using 5-fold cross-validation and an independent Dartmouth-Hitchcock Medical Center (DHMC) cohort (n = 61).

**Results:**

The contrastive multimodal model achieved a c-index of 0.91 (95% confidence interval [CI] = 0.84 to 0.96), significantly outperforming the unimodal models (image-only = 0.76; non-image-only = 0.87) and showing an improvement over the noncontrastive multimodal model (c-index = 0.89), although this difference was not statistically significant. Kaplan-Meier analysis demonstrated clear survival separation across risk strata (log-rank *P *= 4.4 × 10^−5^). Contrastive learning improved representation clustering quality, with silhouette scores increasing from 0.20 to 0.24 (*P* = .05). External evaluation on the DHMC cohort achieved a c-index of 0.87 (95% CI = 0.77 to 0.95) after domain adaptation.

**Conclusion:**

Contrastive multimodal learning significantly enhances survival prediction in grade 2/3 gliomas by effectively integrating histopathology, genomics, and clinical data. This annotation-free approach enables early risk stratification using routinely collected data and shows promise for informing personalized treatment decisions and clinical trial stratification.

## Introduction

Gliomas are the most common primary brain tumors in adults and a major cause of cancer-related morbidity.^1^ Located within the central nervous system, they typically grow infiltratively, making complete surgical resection difficult. The World Health Organization (WHO) classifies gliomas into grades 1-4 based on histopathological features such as proliferation, atypia, necrosis, and microvascular proliferation.^2^ Low-grade gliomas have relatively favorable outcomes, with median survival of ∼7 years,^3^ whereas glioblastomas (grade 4) are highly aggressive, with median survival of ∼15 months.^4-7^ Although glioblastoma outcomes consistently align with histological grade, patients with grade 2/3 gliomas show marked survival heterogeneity despite similar histology, underscoring the need for improved prognostic stratification.^3^^,^^8^

Accurate prognostic assessment is critical for glioma management, guiding treatment, patient counseling, and trial enrollment.^9^ Yet predicting outcomes is difficult due to tumor heterogeneity, spanning molecular profiles, clinical presentations, and imaging phenotypes. Even patients with identical histological diagnoses and treatments often show widely variable survival.^10^^,^^11^ This variability highlights the need for reliable risk stratification tools to distinguish high- from low-risk patients, enabling personalized therapy and reducing the burden of prognostic uncertainty.^12^

Current prognostic models for gliomas incorporate demographic, clinical, histological, and select molecular biomarkers, particularly mutations in isocitrate dehydrogenase (IDH) and O^6^-methylguanine-DNA methyltransferase (MGMT) promoter methylation.^6^^,^^12-17^ Although informative, these factors capture only part of glioma biology. Traditional workflows also depend on manual pathology, which is labor-intensive, time-consuming, and subject to interobserver variability. Moreover, conventional statistical models such as Cox regression assume linear relationships and struggle to integrate the high-dimensional data generated by molecular profiling and digital pathology.^18^

Deep learning offers solutions by automatically extracting complex, nonlinear patterns from multimodal data without manual feature engineering.^19^ Convolutional networks and transformers have achieved strong performance in glioma tasks, including survival prediction and grading, by detecting subtle histopathological features and integrating diverse modalities.^20-22^ These approaches can uncover intricate clinical, imaging, and molecular relationships, potentially surpassing the predictive power of traditional methods.^23^

Contrastive learning has emerged as a powerful approach for representation learning, particularly in medical domains with limited labeled data.^24^^,^^25^ It is a representation learning approach that trains models to bring related samples closer together in the embedding space while pushing unrelated samples farther apart, therefore producing more informative, generalizable representations. In multimodal settings, such as combining histopathology images and genomic data, contrastive learning encourages embeddings from the same patient across different modalities to align while separating embeddings from different patients. This process improves cross-modal consistency and enables the model to learn richer representations that capture complementary biological information. This is especially valuable in medical imaging, where annotated datasets are costly and time-consuming to obtain.^26^

In this study, we developed a multimodal deep learning framework leveraging contrastive learning for survival prediction in grade 2/3 glioma patients. Our approach integrates whole-slide histopathology images, somatic mutation profiles, and clinical-demographic data through a 3-stage training pipeline specifically designed to optimize cross-modal representation alignment and survival prediction accuracy. Unlike previous approaches that rely on single-modality inputs or simple feature concatenation, our framework employs contrastive learning to harmonize heterogeneous data modalities and extract complementary prognostic information. By using only data routinely collected during standard clinical care, our method provides an annotation-free, cost-effective approach to risk stratification that could be readily implemented in clinical practice to support personalized treatment planning and reduce prognostic uncertainty.

## Methods

### Datasets

This retrospective study included 2 cohorts: 498 patients with grade 2/3 glioma from The Cancer Genome Atlas (TCGA) program^27^ and 61 patients treated at Dartmouth-Hitchcock Medical Center (DHMC), a tertiary academic center in Lebanon, New Hampshire, USA. Eligible patients had confirmed WHO grade 2/3 glioma with complete data across modalities: whole-slide histopathology, somatic mutations, demographics, and clinical records including survival outcome. Patients with incomplete survival data or missing modalities were excluded. The TCGA cohort served as the primary training and validation dataset, with somatic mutations data generated using whole-exome sequencing (WES) as described in the TCGA lower-grade glioma consortium study.^28^ The DHMC cohort was used for external evaluation, with mutation data obtained from a targeted next-generation sequencing hotspot panel covering 50 cancer genes.

All data were deidentified before analysis. The use of TCGA data followed TCGA publication policies, and use of DHMC data was approved by the Dartmouth Health Institutional Review Board. Given the retrospective use of deidentified data, informed consent was waived.

### Data preprocessing

Somatic mutation data were converted into binary vectors indicating gene mutation status. Mutations with zero prevalence were excluded, and univariate Cox regression identified survival-associated genes (*P* < .05), yielding 126 mutation features for analysis. Clinical and demographic variables were standardized before model training. Categorical variables were encoded using one-hot encoding, and continuous variables were normalized using *z*-score scaling. Missing values were imputed using cohort means for continuous variables, modes for categorical variables, or explicit “missing” categories when appropriate. Whole-slide images were digitized at 20× (0.5 μm/pixel) or 40× (0.25 μm/pixel) magnification with Leica AT2 or Philips UltraFast scanners; 40× scans were downsampled to 20×. Automated tissue detection identified tumor regions, excluding background and artifacts. Images were split into 224 × 224-pixel patches, with background-dominant patches filtered out.

### Machine learning models

**Figure 1.**
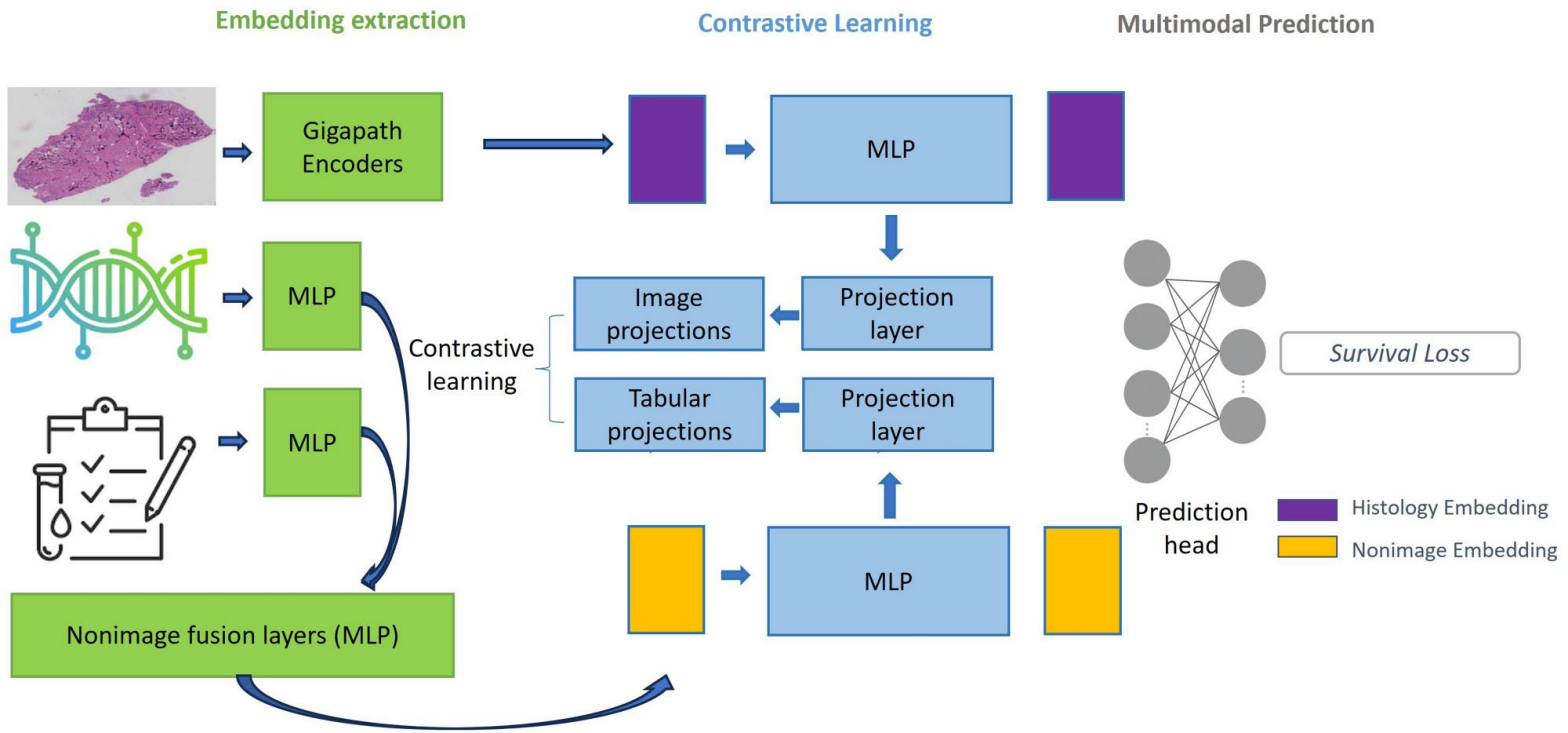
Multimodal contrastive learning framework for glioma survival prediction. Histopathology images are encoded via Prov-GigaPath, whereas genomic and clinical data are processed through multilayer perceptron (MLP) encoders. Contrastive learning aligns cross-modal representations in a shared projection space, and fused embeddings are processed through a prediction head for survival outcome estimation.

The proposed framework uses a multimodal deep learning architecture integrating histopathology images and clinical-genomic data through a 3-stage pipeline. Modality-specific extractors generate embeddings, a contrastive learning module aligns features in a shared latent space, and a fusion component with prediction head estimates survival. This design leverages complementary information across data types while learning unified prognostic representations. An overview of the complete framework architecture and training process is shown in Figure 1, and details of the training pipeline are available in Table S1.

#### Non-image modality

The non-image modality integrates somatic mutations, demographics, and clinical variables using a hierarchical encoding strategy to capture the distinct structures of each data type. Given the high dimensionality and sparse binary nature of mutation data compared with the continuous and categorical clinical variables, we employed separate multilayer perceptron (MLP) encoders to process each data type independently.

The mutation encoder processes binary mutation vectors through fully connected layers with ReLU activations and dropout, whereas the clinical-demographic encoder handles normalized continuous and one-hot categorical variables with similar MLP layers. This parallel design enables each encoder to learn data type-specific representations tailored to genetic and clinical inputs.

Embeddings from both encoders are concatenated and passed through shared fully connected layers to generate unified non-image representations. These shared layers capture interactions between genetic and clinical features while preserving the specialized knowledge from individual encoders, yielding comprehensive patient-level embeddings.

#### Image modality

For histopathology analysis, we used Prov-GigaPath, a whole-slide foundation model with 1.1B parameters based on the LongNet transformer.^29^ Pretrained on 1.3B image tiles from 171 189 slides across 28 cancer centers, it provides robust tissue representations. To adapt for glioma survival prediction, we fine-tuned the model on TCGA using survival-based objectives. Whole-slide images were divided into nonoverlapping 224 × 224 patches at 20× magnification, each encoded with the transformer backbone. Patch-level features were aggregated into slide-level embeddings via LongNet attention, capturing local patterns and global architecture for multimodal survival prediction.

#### Contrastive learning encoders

To align cross-modal representations, we used MLP encoders that project image and non-image embeddings into a shared space for contrastive learning. Each modality passes through separate MLPs with ReLU-activated projection layers, generating aligned representations optimized for similarity learning. The contrastive objective clusters patient-matched embeddings while separating those from different patients, improving feature quality and cross-modal consistency. For final survival prediction, we use the original pre-projection embeddings rather than the projected representations, preserving the modality-specific information while benefiting from the improved alignment learned during contrastive training.

#### Prediction layers

After feature extraction and alignment, pre-projection embeddings from both modalities are concatenated into unified patient representations. These fused embeddings pass through a feed-forward network with ReLU activations and dropout, ending in a prediction layer that outputs a scalar risk score. The model is trained with a negative log partial likelihood loss function, enabling learning of relative risk while handling censored survival data.

### Model training and evaluation

#### Three-stage training pipeline

The framework was trained sequentially across 3 stages. Stage 1 involved independent training of single-modality models using supervised learning. The Prov-GigaPath encoder was fine-tuned for survival prediction using histopathology images, whereas the non-image MLP encoder was trained on demographic, clinical, and mutation data. Both models used the negative log partial likelihood loss to handle censored survival data:


$$\mathrm{Loss}(\theta )=-\frac{1}{N_{\mathrm{event}}}\sum_{i:\mathrm{event}}^{\;}\left({\hat{h}}_{\theta }{(x}_{i})-\log\sum_{j\in R(T_{i})}^{\;}e^{{\hat{h}}_{\theta }{(x}_{j})}\right)$$

where $N_{\mathrm{event}}$ is the number of observed events, ${\hat{h}}_{\theta }{(x}_{i})$ is the predicted risk score, and $R(T_{i})$ represents the set of patients still at risk at time $T_{i}$.

Stage 2 implemented supervised contrastive learning using a combined objective:


Loss(contrastive)=InfoNCE Loss+λ*Negative Log Partial Likelihood Loss

The InfoNCE component maximizes similarity between patient-matched cross-modal embeddings while separating embeddings from different patients. The weighting factor λ balances contrastive alignment with survival-specific optimization.

Stage 3 trained the final prediction layers using concatenated pre-projection embeddings from both modalities, using the same survival loss as Stage 1.

#### Validation and evaluation

We implemented 5-fold cross-validation, in which the dataset is divided into 5 subsets and each subset is used once as a validation set, while the remaining subsets are used for training, to evaluate model performance and reduce overfitting. Splits of folds were kept consistent across all training stages to prevent information leakage. Model performance was evaluated using the concordance index (c-index), and an ensemble was constructed from the best-performing models across folds. Bootstrapping, a resampling technique used to estimate uncertainty in model performance, was used to compute confidence intervals and compare c-index values between models. A t-distributed stochastic neighbor embedding (t-SNE), a dimensionality reduction method for visualizing high-dimensional data, was used to assess clustering of patient representations. Dropout regularization and L2 weight decay were applied throughout training to prevent overfitting.

#### External evaluation

For external validation, we tested the TCGA-trained model on an independent DHMC cohort (n = 61). This posed distributional challenges, mirroring real-world deployment where models must generalize across populations with distinct molecular and demographic profiles. At DHMC, 98% of patients were IDH-mutant vs 77.3% in TCGA, representing a major covariate shift, since IDH status is strongly prognostic. Several TCGA genomic features (eg, aneuploidy scores, mutation burden) were also unavailable in DHMC due to different sequencing methods. These differences created a stringent test of robustness under domain shift. We report both zero-shot performance (no DHMC fine-tuning) and domain-adapted results after fine-tuning on 20% of DHMC for 10 epochs, with evaluation on the remaining 80%. This assessed both out-of-distribution generalization and adaptability with minimal data.

### Feature importance analysis

To interpret multimodal predictions, we applied a 2-step approach focused on non-image features. First, Least Absolute Shrinkage and Selection Operator regression, a regularization method used for feature selection and dimensionality reduction, was applied to identify the most predictive clinical and genomic variables. Next, ordinary least squares (OLS) regression was performed on the selected variables to estimate interpretable coefficients and assess statistical significance. Coefficient magnitudes and directions indicated relative importance and associations with patient survival, whereas Wald *t*-tests identified statistically significant contributions of features.

## Results

### Study samples

We analyzed 498 TCGA grade 2/3 glioma patients and 61 from DHMC. In TCGA, 120 deaths (24.1%) occurred during follow-up. Mean age was 43.0 years (SD = 13.4); 56.0% were male and 92.4% White. Histological diagnoses included astrocytoma in 191 patients (38.4%) and oligodendroglioma in 180 patients (36.1%). In DHMC, 12 deaths (19.7%) occurred. Although demographics were similar, molecular profiles differed: 98% were IDH-mutant vs 77.3% in TCGA. Full characteristics are in Table 1.

**Table 1.** Model performance comparison across different input modalities and training strategies.

Model type	Input modalities	Contrastive learning	C-index (95% CI)	***P*** ^a^
Unimodal	Image only	No	0.76 (0.64 to 0.87)	—
Unimodal	Non-image only	No	0.87 (0.77 to 0.93)	.15^b^
Multimodal	Image + Non-image	No	0.89 (0.82 to 0.95)	.05^b^/.16^c^
Multimodal	Image + Non-image	Yes	0.91 (0.84 to 0.96)	.03^b^/.04^c^

Values represent concordance index with 95% confidence intervals.

a *P* values compare multimodal models with unimodal baselines using bootstrap testing.

b Comparison with non-image only model.

c Comparison with image only model.

### Model performance and comparison between single and multimodal models

Model performance across different input modalities and training strategies is summarized in Table 1. The contrastive multimodal model achieved the highest performance with a c-index of 0.91 (95% CI = 0.84 to 0.96), compared with the noncontrastive multimodal model (c-index = 0.89, 95% CI = 0.82 to 0.95) and both single-modality models: image-only (c-index = 0.76, 95% CI = 0.64 to 0.87) and non-image-only (c-index = 0.87, 95% CI = 0.77 to 0.93). Although the contrastive multimodal model achieved the highest c-index, the confidence intervals overlap with those of the noncontrastive multimodal model, suggesting that the difference between these 2 multimodal approaches should be interpreted cautiously and further validated in larger cohorts. Overall, these results demonstrate the benefit of multimodal integration and suggest that contrastive learning may further enhance performance. Statistical significance testing using bootstrapping confirmed all models outperformed random prediction (c-index = 0.5), serving as a baseline validation. However, pairwise comparisons revealed important differences in the magnitude of improvements. Although the noncontrastive multimodal model showed higher performance than single-modality models, these improvements were not statistically significant (*P* = .16 vs non-image model; *P* = .05 vs image model). In contrast, the contrastive multimodal model achieved statistically significant improvements over both single-modality approaches (*P* = .04 vs non-image model; *P* = .03 vs image model), highlighting the potential benefit of contrastive learning in enabling effective multimodal integration for survival prediction.

### Model capability for risk stratification

**Figure 2.**
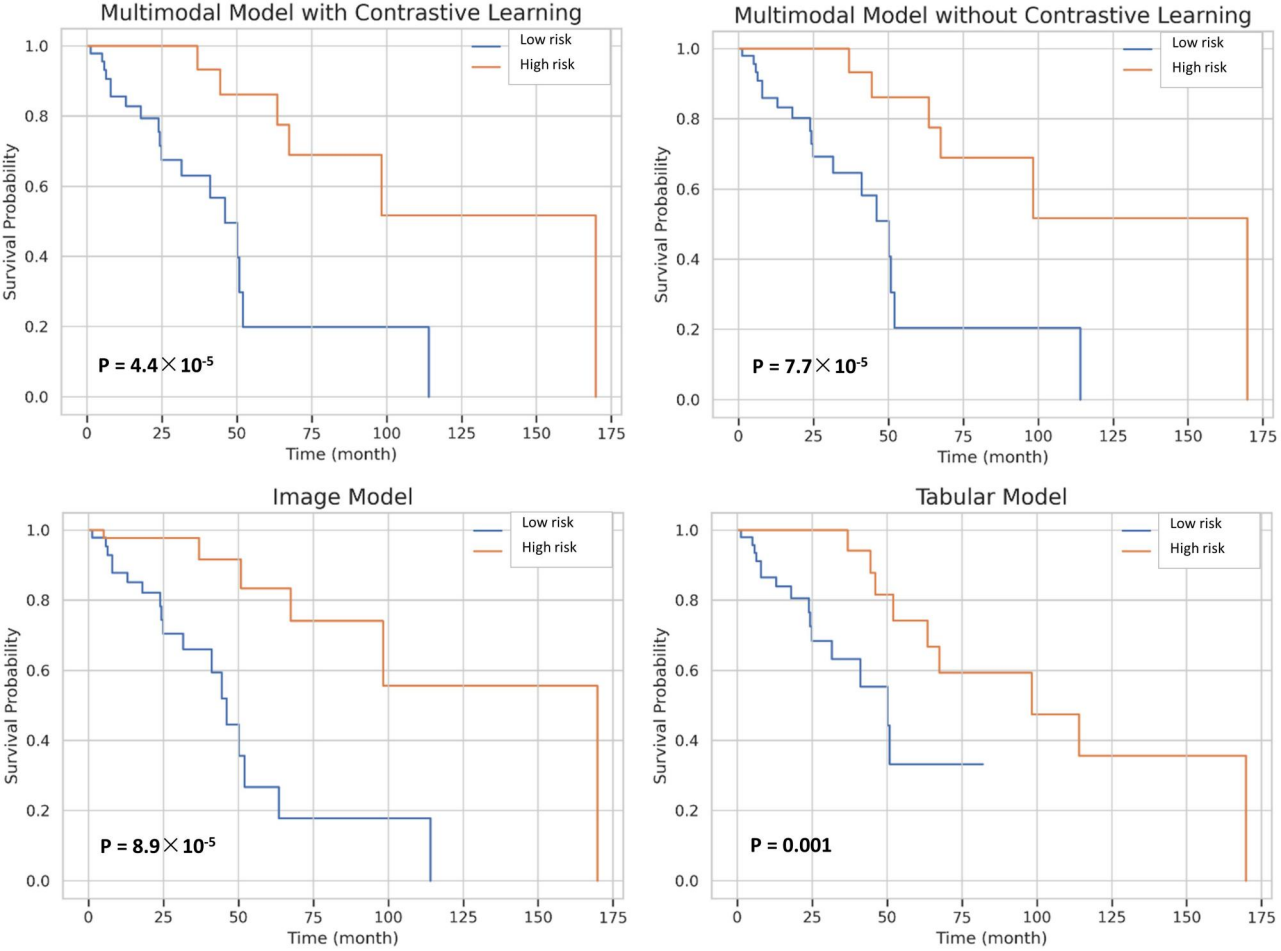
Kaplan-Meier curves showing risk stratification capability of different model approaches. Patients were divided into high-risk and low-risk groups based on median predicted scores. All models demonstrated significant survival separation (log-rank *P* values shown), with the contrastive multimodal model achieving the best discrimination (*P* = 4.4 × 10^−5^).

To evaluate risk stratification, patients were divided into high-risk and low-risk groups based on median predicted scores from each of the 4 evaluated models (image-only, non-image-only, multimodal without contrastive learning, and multimodal with contrastive learning). Kaplan-Meier curves showed clear separation between groups across all models (Figure 2), indicating effective prognostic discrimination. Log-rank tests confirmed significant survival differences between strata for all approaches. The contrastive multimodal model achieved the strongest stratification with a log-rank *P* value of 4.4 × 10^−5^, outperforming the noncontrastive model (*P* = 7.7 × 10^−5^). Both multimodal approaches exceeded single-modality performance, with the non-image model achieving *P* = .0012 and the image-only model *P* = 8.9 × 10^−5^. These results show that multimodal integration enhances stratification, with contrastive learning providing added benefit for identifying patients at different survival risk levels.

### Comparison of learned representations before and after contrastive learning

To evaluate the quality of learned representations, patients were stratified by 5-year survival status and analyzed using t-SNE dimensionality reduction. Comparison of patient representations before and after contrastive learning revealed improved clustering, with patients who died within 5 years showing more compact grouping after contrastive learning. Quantitative assessment was performed using silhouette scores to measure clustering quality based on 5-year survival status. After contrastive learning, the average silhouette score across 5 cross-validation folds increased from 0.20 (SD = 0.03) to 0.24 (SD = 0.06). A paired *t*-test confirmed this improvement was statistically significant (*P* = .05), demonstrating that contrastive learning enhanced the discriminative quality of learned representations for survival prediction.

### External validation and generalizability assessment

External validation was performed on the DHMC cohort (n = 61), which was predominantly IDH-mutant and tested the pipeline’s generalizability under domain shift. To comprehensively assess model robustness, we report both zero-shot performance (without DHMC fine-tuning) and domain-adapted results (after fine-tuning on 20% of DHMC data).

Zero-shot evaluation revealed an improvement of the multimodal models over the single-modality models (c-index 0.69 for multimodal without contrastive learning, 0.64 for multimodal model with contrastive learning encoders, 0.59 for both single-modality models), consistent with the internal validation. However, the contrastive learning encoders did not bring further benefit in addition to multimodal fusion, indicating the need for larger training data to obtain more generalizable contrastive learning encoders. Domain-adapted evaluation confirmed the trends observed in internal validation (Table 2). The noncontrastive multimodal model outperformed both single-modality models (c-index 0.82 vs 0.69 for non-image; 0.82 vs 0.74 for image), although these improvements were not statistically significant (*P* = .11 and *P* = .35, respectively).

**Table 2.** External validation performance on DHMC cohort (n = 61) comparing zero-shot and domain-adapted results.

Model	**Zero-shot C-index (95% CI)** ^a^	**Domain-adapted C-index (95% CI)** ^b^	***P*** ^c^
Non-image only	0.59 (0.40 to 0.76)	0.69 (0.49 to 0.89)	—
Image only	0.59 (0.41 to 0.77)	0.74 (0.58 to 0.86)	.75
Multimodal (no contrastive learning)	0.69 (0.53 to 0.83)	0.82 (0.68 to 0.93)	.11^d^/.35^e^
Multimodal (with contrastive learning)	0.64 (0.47 to 0.79)	0.87 (0.77 to 0.95)	.04^d^/.04^e^

a Zero-shot performance without any DHMC fine-tuning.

b Performance after fine-tuning on 20% of DHMC cohort, evaluated on remaining 80%.

c *P* values compare multimodal models with unimodal baselines using bootstrap testing.

d Comparison with non-image only model.

e Comparison with image only model.

Incorporating contrastive learning further enhanced performance, with the contrastive multimodal model achieving c-index 0.87 compared with 0.82 for the noncontrastive version. The contrastive model significantly outperformed both single-modality approaches (*P* = .04 for both comparisons), confirming the benefit of contrastive learning across different patient populations. These results demonstrate that the pipeline maintains its predictive advantages when applied to data from a different distribution, supporting its potential for clinical deployment with minimal domain adaptation.

### Feature importance analysis

Feature importance analysis of the multimodal model identified 5 key predictors of survival outcomes: IDH1/2 mutations, ADGRG4 mutation, immunotherapy treatment, and EGFR mutation (Table 3, Figure S1). IDH1 and IDH2 mutations showed protective associations with coefficients of −0.08 (95% CI = −0.12 to −0.06) and −0.06 (95% CI = −0.10 to −0.03), respectively, consistent with their established favorable prognostic role in glioma. Conversely, ADGRG4 mutation (coefficient: 0.07, 95% CI = 0.04 to 0.08), immunotherapy treatment (coefficient: 0.06, 95% CI = 0.03 to 0.10), and EGFR mutation (coefficient: 0.05, 95% CI = 0.02 to 0.09) were associated with increased predicted risk. All associations were statistically significant (*P* < .05 by Wald *t*-test), confirming these features as important drivers of the model’s survival predictions.

**Table 3.** Top predictive features identified by LASSO-OLS feature importance analysis.

Feature	**Coefficient (95% CI)** ^a^	*P*
IDH1 mutation	−0.08 (−0.12 to −0.06)	<.0001
IDH2 mutation	−0.06 (−0.10 to −0.03)	<.0001
ADGRG4 mutation	0.07 (0.04 to 0.08)	<.0001
Immunotherapy treatment	0.06 (0.03 to 0.10)	.0007
EGFR mutation	0.05 (0.02 to 0.09)	<.0001

NOTE: CI = confidence interval; EFGR = epidermal growth factor receptor; IDH = isocitrate dehydrogenase; LASSO = Least Absolute Shrinkage and Selection Operator; OLS = ordinary least squares.

a Negative coefficients indicate protective associations; positive coefficients indicate increased risk.

## Discussion

We developed a contrastive learning-enhanced multimodal framework for predicting overall survival in grade 2/3 glioma patients. By integrating histopathology images, somatic mutations, and clinical-demographic data, our model outperformed unimodal and noncontrastive baselines. Unlike prior models relying on late fusion or handcrafted features, our approach leverages a pathology foundation model and contrastive learning to harmonize multimodal embeddings, enabling state-of-the-art discrimination via a fully annotation-free pipeline.^23^

Prognostic uncertainty poses significant challenges in glioma management, where reliable risk stratification is essential for personalized treatment and counseling. Our approach leverages routinely collected data from electronic health records to generate comprehensive patient representations capturing multiple prognostic dimensions. Because the model uses data typically available at diagnosis, it enables early survival estimation without additional clinical burden or invasive procedures.

A key innovation is our use of contrastive learning to align heterogeneous data modalities. By promoting consistency between patient-specific embeddings while reinforcing survival signals through Cox loss, the model learns joint representations optimized for both modality fusion and survival prediction. This enhanced risk-based clustering, as shown by improved silhouette scores, indicates effective harmonization of diverse data into outcome-relevant embeddings.

Multimodal integration consistently outperformed single-modality approaches, supporting the notion that glioma outcomes are best understood through combined histological, molecular, and clinical analysis. External validation on the DHMC cohort confirmed these benefits and demonstrated adaptability to a cohort with shifted feature distribution, requiring minimal fine-tuning, suggesting broad applicability in diverse clinical settings.

Of note, the performance difference between contrastive and noncontrastive multimodal models was modest. This likely reflects the relatively limited dataset size and the already strong predictive signal contained in the clinical and genomic features. In larger datasets or settings with more heterogeneous modalities, contrastive learning may provide greater benefits by enabling more robust cross-modal representation alignment.

IDH1/2 mutations showed protective associations consistent with established favorable prognosis,^30^ whereas EGFR mutations demonstrated increased risk, aligning with their known tumorigenic effects.^31^^,^^32^ ADGRG4 mutations were associated with higher risk, warranting further study, because this adhesion GPCR family member has limited established role in glioma prognosis.^33-35^ These interpretations should be considered cautiously given the retrospective, post hoc nature of the analysis.

Limitations include the moderate sample size (n = 498) and requirement for complete multimodal data, which may restrict broader applicability. Additionally, the external validation was performed on a small cohort with significant distributional differences from the training data. Future work should address missing data scenarios and validate performance in larger, diverse cohorts.

Despite these constraints, our results demonstrate that integrating histopathology, genomics, and clinical data through contrastive multimodal learning enables accurate survival prediction. This scalable, data-efficient approach provides early risk stratification and shows promise for personalized glioma care. With further validation, such models could serve as practical clinical decision support tools, improving individualized glioma management and outcomes.

## Conclusions

We developed a contrastive learning-enhanced multimodal framework for survival prediction in grade 2/3 glioma patients using routinely collected clinical data. By integrating histopathology, somatic mutations, and clinical-demographic features through a 3-stage pipeline, the model achieved high predictive accuracy (c-index = 0.91) and outperformed single-modality baselines while showing a promising trend of improvement over the noncontrastive baseline. Operating without manual feature engineering, the framework supports streamlined clinical use. Contrastive learning was key for generating informative cross-modal patient representations, improving risk-based clustering and survival group discrimination. Validation on the DHMC data confirmed the advantages of multimodal fusion and contrastive learning with minimal fine-tuning, showing adaptability across patient populations. This annotation-free approach enables early risk stratification at diagnosis, supporting personalized treatment planning and clinical trial enrollment decisions. With broader validation, it could serve as a practical decision support tool for precision glioma care.

## Supplementary Material

pkag039_Supplementary_Data

## Data Availability

The TCGA data used in this study are publicly available and can be downloaded from the TCGA website. The DHMC dataset may be made available from the corresponding author upon reasonable request and with appropriate institutional approvals, subject to patient privacy regulations and institutional data sharing policies.

## References

[1] Ostrom QT , Bauchet L, Davis FG, et al The epidemiology of glioma in adults: a state of the science review. Neuro Oncol. 2014;16:896-913. 24842956 10.1093/neuonc/nou087 PMC4057143

[2] Weller M , Wen PY, Chang SM, et al Glioma. Nat Rev Dis Primers. 2024;10:33. 10.1038/s41572-024-00516-y 38724526

[3] Gorovets D , Kannan K, Shen R, et al IDH mutation and neuroglial developmental features define clinically distinct subclasses of lower grade diffuse astrocytic glioma. Clin Cancer Res. 2012;18:2490-2501. 10.1158/1078-0432.CCR-11-2977 22415316

[4] Lerner A , Palmer K, Campion T, et al Gliomas in adults: guidance on investigations, diagnosis, treatment and surveillance. Clin Med (Lond). 2024;24:100240. 39233205 10.1016/j.clinme.2024.100240 PMC11418107

[5] Claus EB , Walsh KM, Wiencke JK, et al Survival and low-grade glioma: the emergence of genetic information. Neurosurg Focus. 2015;38:E6. 10.3171/2014.10.FOCUS12367 PMC4361022 25552286

[6] Noiphithak R , Veerasarn K. Clinical predictors for survival and treatment outcome of high-grade glioma in Prasat Neurological Institute. Asian J Neurosurg. 2017;12:28-33. 10.4103/1793-5482.148791 28413528 PMC5379799

[7] Brown NF , Ottaviani D, Tazare J, et al Survival outcomes and prognostic factors in glioblastoma. Cancers. 2022;14:3161. 10.3390/cancers14133161 35804940 PMC9265012

[8] Jiang S , Zanazzi GJ, Hassanpour S. Predicting prognosis and IDH mutation status for patients with lower-grade gliomas using whole slide images. Sci Rep. 2021;11:16849. 10.1038/s41598-021-95948-x 34413349 PMC8377095

[9] Rapôso C , Vitorino-Araujo JL, Barreto N. Molecular markers of gliomas to predict treatment and prognosis: current state and future directions. Gliomas. Brisbane (AU): Exon Publications; 2021. 10.36255/exonpublications.gliomas.2021.chapter10 34038057

[10] Penkova A , Kuziakova O, Gulaia V, et al Comprehensive clinical assays for molecular diagnostics of gliomas: the current state and future prospects. Front Mol Biosci. 2023;10:1216102. 37908227 10.3389/fmolb.2023.1216102 PMC10613994

[11] Hu LS , Hawkins-Daarud A, Wang L, et al Imaging of intratumoral heterogeneity in high-grade glioma. Cancer Lett. 2020;477:97-106. 32112907 10.1016/j.canlet.2020.02.025 PMC7108976

[12] Liang X , Wang Z, Dai Z, et al Promoting prognostic model application: a review based on gliomas. J Oncol. 2021;2021:7840007. 34394352 10.1155/2021/7840007 PMC8356003

[13] Bell EH , Pugh SL, McElroy JP, et al Molecular-based recursive partitioning analysis model for glioblastoma in the temozolomide era. JAMA Oncol. 2017;3:784. 10.1001/jamaoncol.2016.6020 28097324 PMC5464982

[14] Curran WJ , Scott CB, Horton J, et al Recursive partitioning analysis of prognostic factors in three radiation therapy oncology group malignant glioma trials. J Natl Cancer Inst. 1993;85:704-710. 10.1093/jnci/85.9.704 8478956

[15] Corso CD , Bindra RS, Mehta MP. The role of radiation in treating glioblastoma: here to stay. J Neurooncol. 2017;134:479-485. 28271281 10.1007/s11060-016-2348-x

[16] Franceschi E , Mura A, Lamberti G, et al Concordance between RTOG and EORTC prognostic criteria in low-grade gliomas. Future Oncol. 2019;15:2595-2601. 10.2217/fon-2019-0093 31339049

[17] Geurts M , van den Bent MJ. On high-risk, low-grade glioma: what distinguishes high from low? Cancer. 2019;125:174-176. 30512190 10.1002/cncr.31834 PMC6587541

[18] Jiang N , Wu Y, Li C. Limitations of using COX proportional hazards model in cardiovascular research. Cardiovasc Diabetol. 2024;23:219. 38926821 10.1186/s12933-024-02302-2 PMC11210144

[19] Liu Y , Wu M. Deep learning in precision medicine and focus on glioma. Bioeng Transl Med. 2023;8:e10553. 37693051 10.1002/btm2.10553 PMC10486341

[20] Wu W , Yan J, Zhao Y, et al Multi-task learning for concurrent survival prediction and semi-supervised segmentation of gliomas in brain MRI. Displays. 2023;78:102402. 10.1016/j.displa.2023.102402

[21] Goceri E. Vision transformer based classification of gliomas from histopathological images. Expert Syst Appl. 2024;241:122672. 10.1016/j.eswa.2023.122672

[22] Mobadersany P , Yousefi S, Amgad M, et al Predicting cancer outcomes from histology and genomics using convolutional networks. Proc Natl Acad Sci USA. 2018;115:E2970-E2979. 10.1073/pnas.1717139115 29531073 PMC5879673

[23] Alleman K , Knecht E, Huang J, Zhang LU, Lam S, Decuypere M. Multimodal deep learning-based prognostication in glioma patients: systematic review. Cancers. 2023;15:545. 10.3390/cancers15020545 36672494 PMC9856816

[24] Le-Khac PH , Healy G, Smeaton AF. Contrastive representation learning: a framework and review. IEEE Access. 2020;8:193907-193934. 10.1109/ACCESS.2020.3031549

[25] Liu Z , Alavi A, Li M, Zhang X. Self-supervised contrastive learning for medical time series: a systematic review. Sensors. 2023;23:4221. 37177423 10.3390/s23094221 PMC10181273

[26] Wang WC , Ahn E, Feng D, Kim J. A review of predictive and contrastive self-supervised learning for medical images. Mach Intell Res. 2023;20:483-513.

[27] TCGA Research Network. The Cancer Genome Atlas Program (TCGA). 2023. https://www.cancer.gov/tcga

[28] Brat DJ , Verhaak RG, et al Comprehensive, integrative genomic analysis of diffuse lower-grade gliomas. N Engl J Med. 2015;372:2481-2498. 10.1056/nejmoa1402121 26061751 PMC4530011

[29] Xu H , Usuyama N, Bagga J, et al A whole-slide foundation model for digital pathology from real-world data. Nature. 2024;630:181-188. 10.1038/s41586-024-07441-w 38778098 PMC11153137

[30] Houillier C , Wang X, Kaloshi G, et al IDH1 or IDH2 mutations predict longer survival and response to temozolomide in low-grade gliomas. Neurology. 2010;75:1560-1566. 10.1212/WNL.0b013e3181f96282 20975057

[31] Saadeh FS , Mahfouz R, Assi HI. EGFR as a clinical marker in glioblastomas and other gliomas. Int J Biol Markers. 2018;33:22-32. 10.5301/ijbm.5000301 28885661

[32] Hatanpaa KJ , Burma S, Zhao D, Habib AA. Epidermal growth factor receptor in glioma: signal transduction, neuropathology, imaging, and radioresistance1. Neoplasia. 2010;12:675-684. 20824044 10.1593/neo.10688 PMC2933688

[33] Grady C , Melnick K, Porche K, et al Glioma immunotherapy: advances and challenges for spinal cord gliomas. Neurospine. 2022;19:13-29. 10.14245/ns.2143210.605 35130421 PMC8987559

[34] Yasinjan F , Xing Y, Geng H, et al Immunotherapy: a promising approach for glioma treatment. Front Immunol. 2023;14:1255611. 37744349 10.3389/fimmu.2023.1255611 PMC10512462

[35] Xu S , Tang L, Li X, et al Immunotherapy for glioma: current management and future application. Cancer Lett. 2020;476:1. 32044356 10.1016/j.canlet.2020.02.002

