# Supplementary material for: Contrastive multimodal deep learning for survival prediction in grade 2/3 gliomas
Source: JNCI Cancer Spectr. 2026 Apr 15;10(3):pkag039. doi: 10.1093/jncics/pkag039 (PMC13199061; doi:10.1093/jncics/pkag039)
Supplement: pkag039_Supplementary_Data [file pkag039_supplementary_data.docx]

**Table S1. Training Pipeline Summary**

| **Stage** | **Objective** | **Input** | **Output** |
| --- | --- | --- | --- |
| Stage 1 | Train modality-specific encoders | Image / clinical-genomic data | Image and non-image embeddings |
| Stage 2 | Contrastive alignment + survival optimization | Paired multimodal embeddings | Aligned representations |
| Stage 3 | Survival prediction | Concatenated embeddings | Risk score |

**
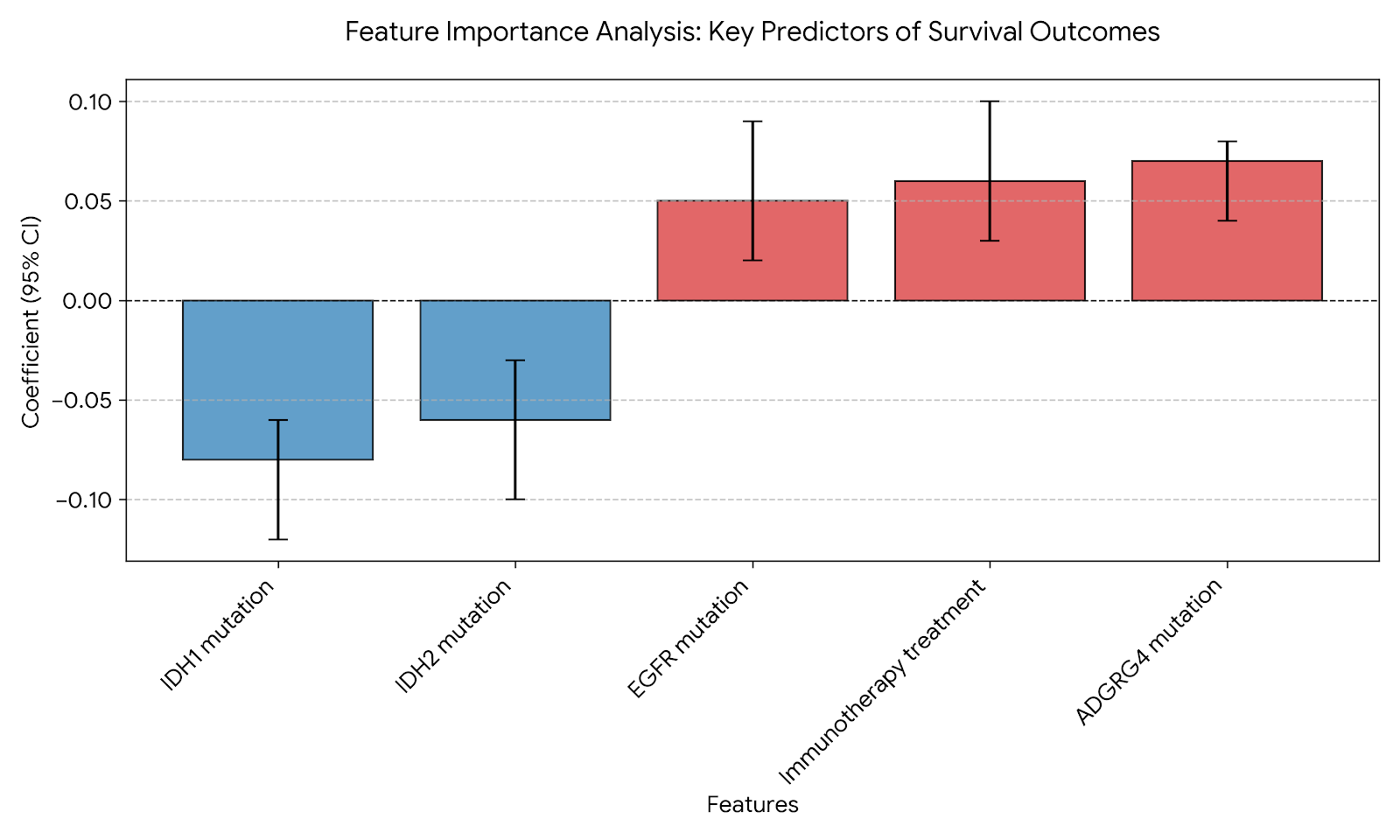
Figure S1. Feature Importance Analysis: Key Predictors of Survival Outcomes**
